# Selected Phenolic Acids Inhibit the Initial Growth of *Ambrosia artemisiifolia* L.

**DOI:** 10.3390/biology11040482

**Published:** 2022-03-22

**Authors:** Maja Šćepanović, Laura Košćak, Valentina Šoštarčić, Laura Pismarović, Ana Milanović-Litre, Kristina Kljak

**Affiliations:** 1 University of Zagreb Faculty of Agriculture, Svetošimunska 25, 10000 Zagreb, Croatia; mscepanovic@agr.hr (M.Š.); vsostarcic@agr.hr (V.Š.); apintar@agr.hr (A.M.-L.); kkljak@agr.hr (K.K.); 2 Institute for Agriculture and Tourism, Karla Huguesa 8, 52440 Poreč, Croatia; laura@iptpo.hr

**Keywords:** common ragweed, phenolic acids, ferulic acid, vanillic acid, *p*-coumaric acid, cover crop

## Abstract

**Simple Summary:**

In the context of international commitments to reduce the environmental impact of herbicides, ecologically more favorable control method approaches must be explored. This is particularly important for allergenic *Ambrosia artemisiifolia* L., one of the most harmful species in the world. Secondary plant metabolites and, in particular, some phenolic compounds are known to have a strong allelopathic effect on weed growth. In this study we investigated whether phenolic acids (chlorogenic acid, caffeic acid, ferulic acid, gallic acid, protocatechuic acid, *p*-hydroxybenzoic acid, syringic acid, vanillic acid, and *p*-coumaric acid) can inhibit the early growth of *A. artemisiifolia*. Phenolic acids were tested at five different dose levels that were up to 16 times than those naturally occurring in plants. The results show that the suppression of the early growth of *A. artemisiifolia* is strongly dependent on phenolic acid and its dose. Treating seeds with ferulic acid, vanillic acid, *p*-coumaric acid, *p*-hydroxybenzoic acid, or a mixture of all phenolic acids resulted in significantly better inhibition of early growth parameters than other phenolic acids. However, none of the phenolic acids tested were effective as bioherbicides at their naturally occurring doses in plants. Therefore, selected doses of phenolic acids with significantly reduced herbicide doses should be further explored to effectively control *A. artemisiifolia*.

**Abstract:**

This study aimed to investigate whether different doses of specific phenolic acids (chlorogenic acid, caffeic acid, ferulic acid, gallic acid, protocatechuic acid, *p*-hydroxybenzoic acid, syringic acid, vanillic acid, and *p*-coumaric acid), alone or in combination, can inhibit the early growth of the common ragweed (*Ambrosia artemisiifolia* L., Asterales: Asteraceae). A seed bioassay was performed in Petri dishes and placed in a climate chamber to assess the effects of five dose levels of phenolic acids to radicle and shoot length, as well seedling biomass of *A. artemisiifolia*. The lowest dose of phenolic acid corresponded to the natural phenolic acid concentration previously reported in dry plant tissue samples from Brassicaceae cover crop plants. Results show that the inhibition of the early growth of *A. artemisiifolia* depends strongly on phenolic acid. Across different treatments, high doses of phenolic acids significantly shortened shoots and radicles, as well as reduced seedling biomass. Treating seeds with ferulic acid alone, vanillic acid alone, *p*-hydroxybenzoic acid alone, or a mixture of all phenolic acids significantly reduced all early growth parameters. The estimated effective dose for the 50% inhibition (ED_50_) of radicle growth in *A. artemisiifolia* seedlings was 368.39 ± 59.85 × 10^−8^ mol with ferulic acid, 135.41 ± 17.65 × 10^−8^ mol with *p*-coumaric acid, 810.36 ± 134.15 × 10^−8^ mol with *p*-hydroxybenzoic acid, and 160.11 ± 12.30 × 10^−8^ mol with the combination of all phenolic acids.

## 1. Introduction

Secondary plant metabolites that are released into the environment can help or harm vegetation growth and development [1]. The allelopathic effects of phenols, a major group of plant allelochemicals [2], on cell expansion, membrane permeability, nutrient uptake, photosynthesis, chlorophyll synthesis, and enzymatic activity have been reported [3]. Certain phenolic compounds can also exert allelopathic effects at the early seedling stage [3,4], particularly for non-weedy species [5]. For example, catechin, syringic acid, and rutin can significantly reduce the seedling growth of weeds such as *Corchorus olitorius* L. [6], while *p*-hydroxybenzoic acid, protocatechuic acid, and vanillic acid can reduce the initial growth of *Echinochloa crus-galli* L. (P. Beuv.) and *Galinsoga parviflora* Cav. [7]. *Brassica nigra* L. inhibits the growth of other weed species by producing caffeic acid, syringic acid, and *p*-coumaric acid [2]. Salicylic acid, ferulic acid, hydroxybenzoic acid, and hydroxyphenyl acetic acid have shown allelopathic effects on *Avena fatua* L. [8]. Certain plant species may act as environmentally friendly herbicides by producing phenolic compounds that inhibit weed growth [2,7,9].

Members of the Brassicaceae family are known to produce high concentrations of biologically active compounds [10,11,12], and they are often grown as cover crops. In fact, plants belonging to this family have recently emerged as an alternative to herbicides given their ability to inhibit the germination and growth of weeds [10]. Therefore, these species are considered to be critically important in the context of the European Green Deal and international commitments to reduce the environmental impact of herbicides [13]. The target specificity and rapid degradation of bioherbicides in the environment require more attention in the development of commercial products [14]. However, to effectively use cover crop species as bioherbicides, it is necessary to specify which type of phenolic compounds is responsible for weed growth inhibition.

In previous work, aqueous extracts of certain Brassicaceae cover crop species inhibited the germination and early growth of the common ragweed, *Ambrosia artemisiifolia* [12], one of the most harmful plant species in the world [15]. In the continental parts of Croatia, *A. artemisiifolia* is the most abundant weed species affecting the summer crops [16]. Moreover, it is considered to be a highly allergenic species around the world. Croatia is one of the three European countries with the highest density of pollen and seeds per area produced by such uncontrolled plants [17]. This allergenic species spreads rapidly along roadsides, where it is often controlled by mowing. An effective mowing regime is critical to reduce the spread of the plant seeds [18]. On the other hand, in almost all arable crops in Croatia, this species is controlled with herbicides [19]. However, resistance to several herbicide groups has been developed [20], and recently the first case of *A. artemisiifolia* resistance to ALS herbicides was detected in Croatia [21]. The decreasing number of herbicides available on the European market together, with social demands towards pesticide-free agriculture, require other approaches to eliminate *A. artemisiifolia* from the cultivated landscape.

It has been hypothesized that Brassicaceae cover crops inhibit *A. artemisiifolia* by producing phenolic acids, detected in dry plant tissue samples [12]. Using such phenolic compounds may be an effective method to eliminate these weeds from cultivated landscapes [8], especially since *A. artemisiifolia* has developed resistance to various types of synthetic herbicides [20]. We are unaware of reports on the effects of phenolic compounds on the early growth of *A. artemisiifolia*. Therefore, in this study, we investigated whether various phenolic compounds, when applied alone or as a mixture of all, can inhibit the germination and early growth of *A. artemisiifolia.* These phenolic compounds were previously identified and quantified from aqueous extracts of certain Brassicaceae cover crop species using liquid chromatography–mass spectrometry [12]. We wanted to test not only single compounds but also their combinations, because they have been shown to interact synergistically in the field [22].

Thus, the aim of this laboratory study was to evaluate the inhibitory potential of five different doses of *p*-coumaric acid, chlorogenic acid, caffeic acid, ferulic acid, gallic acid, protocatechuic acid, *p*-hydroxybenzoic acid, syringic acid, and vanillic acid applied alone and all together as a mixture of phenolic acids toward the early growth of *A. artemisiifolia.* In accordance with the main aim, an additional aim of the study was to estimate the dose of phenolic acids required to inhibit the radicle growth of *A. artemisiifolia* by 50% (ED_50_).

## 2. Materials and Methods

### 2.1. Seed Collection

In October 2018, mature seeds of *A. artemisiifolia* were collected from single-plant populations maintained at the Šašinovec Experimental Station (45°51′05.2″ N, 16°10′34.1″ E) at the University of Zagreb Faculty of Agriculture, Croatia. Seeds were cleaned and stored in paper bags at 4 °C. Using a stereomicroscope, seeds of uniform size and color that had no visible signs of being eaten by insects were selected for experiments. Seeds were used in experiments only if a prior germination test showed that >70% of the tested seeds germinated.

### 2.2. Seed Bioassay

Different doses of chlorogenic acid, caffeic acid, ferulic acid, gallic acid, protocatechuic acid, *p*-hydroxybenzoic acid, syringic acid, vanillic acid, and *p*-coumaric acid, individually or all together (a mixture of all 9 phenolic acids in doses of each corresponding to individual doses), were dissolved in distilled water (100 mL) and sonicated at 35 kHz and 80 °C (Sonorex TK 52, Bandelin, Germany) in order to obtain homogeneous solutions at five different concentrations (*w*/*v*). The lowest concentration corresponded to the natural phenolic concentration found in dry plant tissue samples of cover crop plants (*Sinapis alba, Raphanus sativus* and *Camellina sativa*): gallic acid 65.5 µg/g, caffeic acid 102.5 µg/g, ferulic acid 276 µg/g, vanillic acid 79.3 µg/g, syringic acid 27.3 µg/g, *p*-hydroxybenzoic acid 222.3 µg/g, protocatechuic acid 100.5 µg/g, chlorogenic acid 100 µg/g, and *p*-coumaric acid 84.5 µg/g, and the aqueous extract was prepared as described in [21]. These concentrations were chosen following the approach of using the highest detected phenolic concentration among these three Brassicaceae cover crop species. To compare the efficacy of the phenolic acids on the early growth of *A. artemisiifolia*, the doses of phenolic acids (mol) were calculated from the previously mentioned concentrations to obtain the amount of bioactive substance in the volume of solution used; only ferulic acid differed, as ½ of the previously mentioned concentration was used. The other doses were two, four, eight, or 16 times this lowest dose (Table 1).

Prior to performing the seed bioassays, *A. artemisiifolia* seeds were soaked in a 2% KNO_3_ solution for 24 h to break dormancy. For each of the treatments, a total of 25 seeds were placed on filter paper in a Petri dish (90 mm diameter), after which 5 mL of a specific phenolic acid or mixture of phenolic acids at a specific dose were added. Distilled water (5 mL per Petri dish) was added as a control treatment. Each treatment was replicated in four replicates, and replicates were assigned to one of the phenolic acid dose levels in a complete randomized block scheme. The dishes were placed in a climate chamber (HPP 108, Memmert, Germany) under the following conditions: photoperiod, 12 h/12 h; day temperature, 25 °C; night temperature, 15 °C; humidity, 70%; and light intensity, 40–50 μmol/m^2^ (LED light). All dishes were hermetically sealed with Parafilm to prevent evaporation. After two weeks, the early growth of seedlings was measured based on shoot length, radicle length, and seedling biomass. Seeds were classified as having germinated if their radicle length was >1 mm.

The inhibition percentage, reflected through radicle and shoot length, as well as seedling biomass, was calculated using the formula:

% inhibition = [(Xc − Xt)/Xc] × 100

where Xc—% seedling length and biomass in control and Xt—% seedling length and biomass in treatment with phenolic acids.

### 2.3. Statistical Analysis

The normality of the data and the homogeneity of variance using the Kolmogorov–Smirnov test and Levene’s test were tested. Inter-group differences were assessed by two-stage (hierarchically) nested analysis of variance with dose level nested in levels of the main factor—phenolic acid—using the MIXED procedure in SAS 9.4 (SAS Institute, Cary, NC, USA). Differences that were significant based on the F-test were confirmed as significant using Tukey’s test for means of phenolic acid and means of dose level. Differences associated with *p* < 0.05 were considered significant. Data on the reduction of radicle length in *A. artemisiifolia* seedlings were subjected to non-linear regression using the *drc* package in R (based on Ritz et al. [23]). From this analysis, the median effective dose (ED_50_) was calculated, defined as the dose of phenolic acid resulting in a 50% reduction in seedling radicle length.

## 3. Results

Each of the nine phenolic acids and the combination of all of them exerted quite different effects on the early growth of *A. artemisiifolia* seedlings. Furthermore, we observed a significant interaction between the type of phenolic acid used and its dose, indicating that different doses of phenolic acids had quite different effects on the early growth of *A. artemisiifolia* seedlings (Table 2).

Our results show that phenolic acids differed substantially in their ability to inhibit shoot growth, seedling, and biomass (Table 1). At the lowest dose level (D1), there were no significant differences among any of the phenolic acids. As dose level increased, all phenolic acids except gallic acid, caffeic acid, and syringic acid showed greater inhibition of early growth. Indeed, at the highest dose level (D5), all phenolic acids showed the maximal inhibition of early growth, while caffeic acid and syringic acid inhibited growth by no more than 25%.

Data are reported as mean ± standard error. The experiment was performed twice with four replicates each time. The early growth parameters of *A. artemisiifolia* measured on control are: radicle length = 3.31 cm, shoot length = 4.48, and fresh seedling biomass = 0.2976 g. Differences between different doses of a particular phenolic acid or between the same doses of different phenolic acids were tested for significance using two-stage nested analysis of variance, wherein the factor dose was nested in the main factor (phenolic acid). Values with different lowercase letters (a–c) for the same phenolic acid or with different uppercase letters (A–F) between different phenolic acids differ significantly based on Tukey’s least significant difference test (*p* < 0.05).

*A. artemisiifolia* growth was strongly inhibited by ferulic acid at a dose of 568.54 × 10^−8^ mol (D4), vanillic acid at a dose of 377.3 × 10^−8^ mol (D5), and the phenolic acid mixture at a concentration of 343.85 × 10^−8^ mol (D5). Compared to ferulic acid and vanillic acid, the phenolic acid mixture caused greater reduction in shoot growth (92.2% vs. 73.5% and 86%) and radicle length (98.8% vs. 76% and 96.3%), as well as biomass (88.5% vs. 60.7% and 71.9%). In addition, *p*-hydroxybenzoic acid and *p*-coumaric acid showed significant inhibitory potential against the early growth of *A. artemisiifolia*, although the reductions were smaller (≤65%) than with ferulic acid, vanillic acid, or the mixture. These findings suggest that vanillic acid, *p*-hydroxybenzoic acid, *p*-coumaric acid, and the phenolic acid mixture inhibited the early germination of *A. artemisiifolia*, but only at the highest dose (D5). Ferulic acid, in contrast, showed inhibitory effects even at a lower dose level. At 568.54 × 10^−8^ mol (D4), ferulic acid inhibited germination by 60–76%, similar to the inhibition by the phenolic acid mixture. Only at D5 did *p*-hydroxybenzoic acid inhibit growth parameters to a similar extent: shoot growth by 56.9%, radicle length by 65%, and biomass by 47.7%.

However, if the doses of the phenolic acids within the dose level were compared, the highest dose level of vanillic acid and *p*-coumaric acid were 1.5 and almost 4 times lower, respectively, than the highest dose level of ferulic acid. Considering that the 4-fold lower dose of *p*-coumaric acid (compared to ferulic acid) inhibited the early growth parameters of *A. artemisiifolia* by 49–59%, it is reasonable to assume that the response would be even stronger if higher doses of *p*-coumaric acid were used.

**Figure 1:**
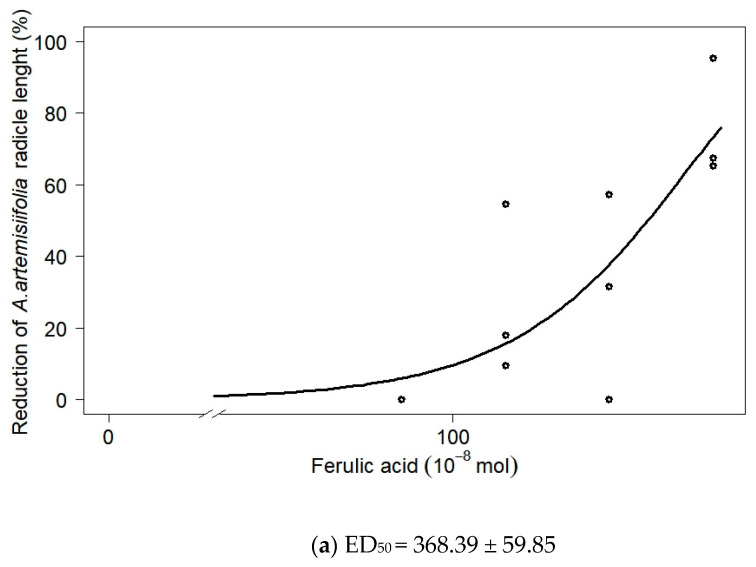

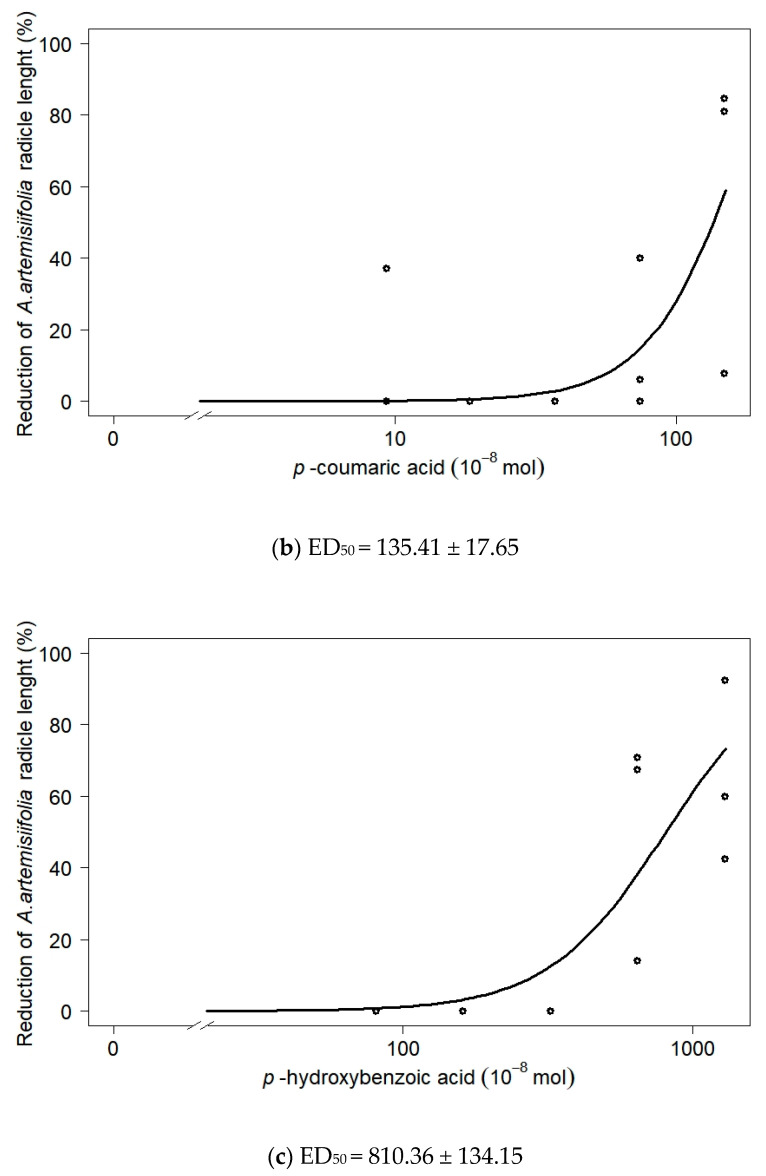

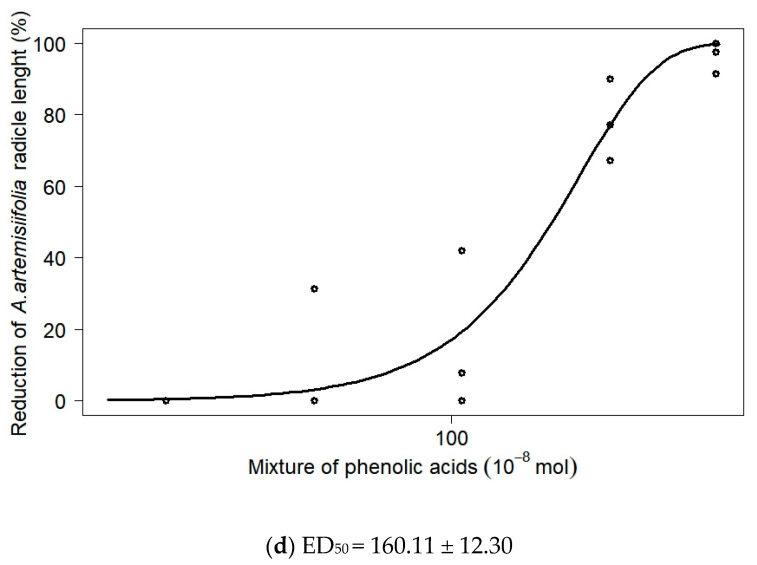
Log-logistic dose–response curve depicting radicle length reduction in *A. artemisiifolia* seedlings when treated with (**a**) ferulic acid, (**b**) *p*-coumaric acid, (**c**) *p*-hydroxybenzoic acid, or (**d**) mixture of all nine phenolic acids (estimated value ± standard error, ×10^−8^ mol). The dots represent observed data, while the solid curve represents the response predicted by non-linear regression.

The dose–response curves were calculated for ferulic acid, *p*-coumaric acid, *p*-hydroxybenzoic acid, and the phenolic acid mixture (Figure 1a–d). The estimated dose ± standard error of phenolic acid required to inhibit radicle growth by 50% (ED_50_, × 10^−8^ mol) was 368.39 ± 59.85 for ferulic acid, 135.41 ± 17.65 for *p*-coumaric acid, 810.36 ± 134.15 for *p*-hydroxybenzoic acid, and 160.11 ± 12.30 for the phenolic acid mixture. These values correspond to 5.2, 14.5, 10, or 6 times the respective doses of these compounds naturally occurring in the dry plant tissue of cover crop species.

## 4. Discussion

Higher doses of almost all phenolic acids (except gallic acid, syringic acid, and chlorogenic acid) inhibited the early growth of *A. artemisiifolia* more effectively than lower doses, which is consistent with previous results [24,25]. Similarly, when aqueous extracts of Brassicaceae cover crop species were used in bioassay, it was found that the early growth inhibition of *A. artemisiifolia* was possible only at the highest concentrations (0.1 g cover crop powder per mL). Although weed species may vary in their sensitivity to allelochemicals [26], it seems unlikely that allelochemicals completely inhibit weed growth [14].

Our results are consistent with several studies on the effects of phenolic compounds on crops and weedy species. Ferulic acid and *p*-coumaric acid reduce leaf water potential and stomatal diffusion in *Sorghum bicolor* L. [22]. Guenzi and McCalla [27] estimated that residue from a single *S. bicolor* crop can add about 100 kg/ha of *p*-coumaric acid to the soil. Furthermore, *p*-coumaric and ferulic acid at 1 mmol/L had phytotoxic effects on *Amaranthus retroflexus* and *Digitaria sanguinalis* and their physiological processes [28].

Although several reports have proven that these phenolic acids inhibit weed growth, very few studies have been conducted to identify the mode of action and physiological changes in weeds [24]. However, phenolic compounds in herbal extracts have been found to reduce amylase activity in weeds, which delays seed germination due to a slow starch hydrolysis process [29]. Moreover, an increase in lipid globules, decrease in mitochondria and the destruction of mitochondrial and nuclear membranes in weeds were observed during bioherbicide treatment [30].

Furthermore, the additive or synergistic inhibitory effects of such allelopathic compounds may be more detrimental than the effects of the individual compounds, even at lower doses [22]. Similarly, the present study showed that the mixture of phenolic acids also had the best inhibitory effect on *A. artemisiifolia*. But our results also highlighted the great inhibitory potential of ferulic acid for *A. artemisiifolia* which, to our knowledge, has not been shown before.

Among the phenolic acids naturally occurring in plants, the most persistent appear to be ferulic acid, *p*-coumaric acid, vanillic acid, and *p*-hydroxybenzoic acid [31]. In the present study, we found that these are the phenolic acids that had significant effects on the growth and germination of *A. artemisiifolia*. Several factors may reduce their inhibitory strength in the field. After entering the soil, soil microorganisms may convert or utilize phenolic compounds, reducing their biological activities [32]. Furthermore, certain phenols such as *p*-coumaric acid, protocatechuic acid, or caffeic acid are less likely to be taken up by weeds because they easily adsorb onto clay minerals and form chelate complexes with metals in the soil [2]. In no-till systems, the concentrations of individual phenolic acids in soil are far below the levels required for growth inhibition in vitro [33]. On the other hand, if allelopathic cover crops are used to suppress weeds in the field [4,6,11,12], optimal agricultural practices or pedoclimatic conditions are critical for effective weed control. For example, a later sowing date may result in less suppression of weeds because less biomass is produced [34] or glucosinolate concentration in plant tissues decreases from germination to plant growth [35]. Additionally, the weed suppression ability of cover crop species depends on sufficient precipitation for germination and growth [36]. Furthermore, the intensity of the allelopathic effect in the field depends on the different transformations that the organic compounds will suffer after release to the environment [37].

Accordingly, inhibitory phenolic compounds may need to be applied directly to the leaves of the target weeds in order to be effective i.e., to be used as bioherbicides [14]. Since there are no data in the literature on the behavior of phenolic acids on *A. artemisiifolia,* we compared similar studies with other weed species. Indeed, the photosynthetic efficiency of *Rumex acetosa* was significantly reduced when seedlings were irrigated with 1.5 mmol ferulic acid and *p*-hydroxybenzoic acid [37].

In present study, the initial concentration of each tested phenolic acid corresponded to the natural phenolic concentration of the plants tested in the field trial, as earlier suggested [7]). On the other hand, in another study [25], mostly much higher doses of phenolic acids were used, but the authors reported very weak or almost no allelopathic activity from these acids in six different weed species. Specifically, *A. artemisiifolia* may be less sensitive to allelochemicals [12], which is consistent with the idea that weed species differ in their sensitivity to allelochemicals due to differences in seed size [38]. and *A. artemisiifolia* has a mass weight about 10–15 times higher than many weed species [39]. Therefore, it is less likely that lower doses of phenolic acids could serve as potential bioherbicides.

Our study showed that ferulic acid, *p*-hydroxybenzoic acid, vanillic acid, and *p*-coumaric acid could suppress the early growth of *A. artemisiifolia* only when administered at higher doses than occur in plant tissues. Therefore, in order to use them as bioherbicides, the interaction of phenolic acids with soil microbial composition must be tested, as negative or positive feedback on plant growth has previously been found [25,40].

Our work suggests that these phenolic compounds cannot serve as effective herbicides at their naturally occurring concentrations in plants. The efficacy of allelochemicals has been already shown to be weak when used alone, but they become more effective when combined with other integrated weed management strategies [14]. Thus, combining selected phenolic compounds with reduced levels of synthetic chemical herbicides may provide effective weed control [41,42]. Further research should be conducted to test this approach using *A. artemisiifolia*. Based on the results of the present study, the combined effect of ferulic acid, *p*-hydroxybenzoic acid, vanillic acid, and *p*-coumaric acid and reduced doses of herbicides on *A. artemisiifolia* should be tested. Certainly, the economical perspective of utilizing allelochemicals for herbicidal purpose should also be considered when choosing this approach to control *A. artemisiifoilia*. The phenolic acids used in the present study were obtained from Sigma-Aldrich^®^ (Waltham, MA, USA) as an analytical standard with high purity. We can estimate the potential financial costs of using phenolic acids based on the ED_50_ for 50% radicle reduction (Figure 1). For example, the cost of ferulic acid applied to one hectare at a spray rate of 200 L/ha is approximately €35 for each phenolic acid. The cost of the herbicide Adengo^®^ (Bayer Crop Science, Leverkusen, Germany), which is widely used to control *A. artemisiifolia* in maize fields in Croatia, is about 100 €/ha. The possibility of reducing the herbicide dose by adding phenolic acids could therefore also be an economical prospect for agricultural producers.

In addition, further studies are required to investigate the possible negative effects of selected phenolic compounds on arable crops [12], since the current literature on this subject remains contradictory. For example, Krogmeir and Bremner [31] stated that phenolic acids do not affect the seedling growth of maize, while Devi and Prasad [43] concluded that ferulic acid notably decreased radicle length and fresh biomass of maize. Similarly, Patterson [44] reported that *p*-coumaric and ferulic acid at 10^−3^ mol severely reduced the net photosynthetic rate and the stomatal conductance of fully expanded soybean leaves.

Even if phenolic compounds usually have much shorter half-lives in the environment than synthetic compounds [45], examining their potential off-target effects is important, particularly if they are to be used against problematic weed species such as *A. artemisiifolia.*

## 5. Conclusions

The present study demonstrates the potential of ferulic acid, *p*-coumaric acid, vanillic acid, and *p*-hydroxybenzoic acid to inhibit the early growth of *A. artemisiifolia*. Further studies should test *p*-coumaric acid at higher doses. Because several factors can reduce allelochemical inhibitory power in the field, those phenolic acids could be used as foliar bioherbicides. However, none of these phenolic acids can serve as effective herbicides in their naturally occurring concentrations in crops; the inhibitory phenolic acids may need to be applied in combination with significantly reduced herbicide doses to effectively control *A. artemisiifolia*. Based on the estimated ED_50_ value, for a 50% reduction of radicle of *A. artemisiifolia*, 368 × 10^−8^ mol ferulic acid, 135 × 10^−8^ mol *p*-coumaric acid and 810 × 10^−8^ mol *p*-hydroxybenzoic acid could be mixed with certain synthetic herbicides in further studies. Namely, despite the great interest in the extraction of allelopathic compounds, very few plant-based bioherbicides are available for commercial use [45].

## Figures and Tables

**Table 1:** Inhibition of the early growth of *Ambrosia artemisiifolia* by phenolic acids at different dose levels.

Phenolic Acid	Dose *	×10^−8^ mol	Inhibition Rate (%)		
			Shoot	Radicle	Biomass
Gallic acid	D1	19.3	0.3 ± 0.3 Aa	4.6 ± 4.6 Ba	0.9 ± 0.9 Ba
	D2	38.5	0.0 ± 0.0 Ca	0.0 ± 0.0 Ca	1.8 ± 1.8 Ba
	D3	77.0	0.0 ± 0.0 Da	0.2 ± 0.2 Ca	1.2 ± 0.7 Ba
	D4	154.0	0.0 ± 0.0 Da	0.0 ± 0.0 Ca	6.5 ± 3.4 Ca
	D5	308.0	1.4 ± 1.4 Fa	14.2 ± 7.1 Da	11.1 ± 3.8 DEa
Caffeic acid	D1	28.4	0.3 ± 0.3 Aa	0.0 ± 0.0 Ba	14.2 ± 2.5 ABab
	D2	56.9	0.1 ± 0.1 BCa	0.0 ± 0.0 Ca	1.8 ± 1.3 Bc
	D3	113.8	0.0 ± 0.0 BCa	0.2 ± 0.2 Ca	10.2 ± 5.4 Bab
	D4	227.6	0.0 ± 0.0 Da	0.0 ± 0.0 Ca	19.8 ± 5.1 Ca
	D5	455.2	1.4 ± 1.4 DEa	8.2 ± 8.2 Da	25.3 ± 11.8 DEa
Ferulic acid **	D1	71.07	3.4 ± 3. Ac	0.0 ± 0.0 Bc	10.8 ± 9.1 ABc
	D2	142.14	28.6 ± 12.9 Ab	27.4 ± 13.9 Ab	37.6 ± 2.6 Ab
	D3	284.27	37.89 ± 8.3 Ab	29.6 ± 16.5 Ab	36.3 ± 5.3 Ab
	D4	568.54	73.47 ± 9.2 Aa	76.1 ± 9.0 Aa	60.7 ± 8.8 Aa
Vanillic acid	D1	23.6	8.4 ± 8.4 Ab	4.2 ± 1.0 Ab	6.71 ± 3.36 AB
	D2	47.2	0.0 ± 0.0 Cb	6.6 ± 6.6 ABb	9.56 ± 6.37 Bb
	D3	94.3	0.2 ± 0.2 Db	2.8 ± 2.8 Cb	2.33 ± 2.33 Cb
	D4	188.6	0.0 ± 0.0 Db	13.5 ± 7.2 Cb	2.65 ± 2.65 Db
	D5	377.3	86.0 ± 0.9 ABa	98.8 ± 0.3 Aa	71.9 ± 2.6 Aba
Syringic acid	D1	6.9	16.5 ± 6.1 Aa	0.0 ± 0.0 Ba	20.7 ± 6.0 Aa
	D2	13.8	12.7 ± 4.4 Aba	0.0 ± 0.0 Ca	18.9 ± 9.7 Ba
	D3	27.6	1.9 ± 1.4 Da	0.0 ± 0.0 Ca	10.3 ± 6.9 Ba
	D4	55.1	6.3 ± 6.3 Da	0.0 ± 0.0 Ca	9.0 ± 7.2 Ca
	D5	110.2	7.6 ± 7.6 DEa	1.6 ± 1.6 Da	20.2 ± 7.87 DEa
*p*-hydroxybenzoic acid	D1	80.5	0.6 ± 0.6 Ab	0.0 ± 0.0 Bb	7.7 ± 4.4 ABb
	D2	160.9	6.6 ± 6.6 Cb	0.0 ± 0.0 Cb	11.7 ± 5.9 Bb
	D3	321.9	9.1 ± 3.5 BCb	0.0 ± 0.0 Cb	16.8 ± 6.7 Bb
	D4	643.8	46.5 ± 10.9 Ba	50.7 ± 18.3 Ba	41.9 ± 4.7 Ba
	D5	1287.6	56.9 ± 15.3 Ca	64.9 ± 14.6 BCa	47.7 ± 8.5 Ca
Protocatechuic acid	D1	32.6	3.7 ± (1.9) Aab	0.0 ± 0.0 Ba	7.7 ± 1.4 ABa
	D2	65.2	1.7 ± (1.7) Cc	0.0 ± 0.0 Ca	10.9 ± 6.2 Bc
	D3	130.4	13.1 ± 7.2 BCab	8.9 ± 5.2 ABa	17.5 ± 2.1 Bab
	D4	260.8	4.6 ± 1.8 Dab	3.3 ± 1.6 Ca	8.3 ± 5.3 Cc
	D5	521.7	19.7 ± 3.0 Da	20.9 ± 13.0 Da	29.0 ± 7.0 Da
Chlorogenic acid	D1	14.1	5.4 ± 3.5 Aa	0.0 ± 0.0 Ba	13.0 ± 3.0 ABab
	D2	28.2	6.9 ± 6.9 Ba	0.3 ± 0.3 Ca	13.2 ± 10.3 Bab
	D3	56.5	9.3 ± 9.30 BCa	0.0 ± 0.0 Ca	18.2 ± 10.0 Bab
	D4	113.0	7.5 ± 7.1 Da	0.6 ± 0.6 Ca	5.5 ± 5.3 Cc
	D5	225.9	20.2 ± 4.7 Da	5.9 ± 5.9 Da	23.2 ± 6.6 DEa
*p*-coumaric acid	D1	9.3	0.0 ± 0.0 Ab	12.4 ± 12.4 Bb	1.5 ± 1.4 Bb
	D2	18.5	0.2 ± 0.2 Cb	0.0 ± 0.0 Cb	5.1 ± 2.6 Bb
	D3	37.0	4.4 ± 4.4 BCb	0.0 ± 0.0 Cb	2.1 ± 2.1 Bb
	D4	74.1	8.1 ± 4.2 Da	15.4 ± 12.5 Cb	16.6 ± 8.3 Cb
	D5	148.2	59.9 ± 13.5 Ca	57.8 ± 25.0 Da	49.4 ± 5.8 Ca
Mixture of all phenolic acids	D1	26.27	12.6 ± 3.7 Ac	0.0 ± 0.0 Bb	5.6 ± 5.6 ABc
	D2	52.54	11.1 ± 11.1 Bc	10.4 ± 10.4 ABb	3.4 ± 3.4 Bc
	D3	105.09	19.8 ± 8.4 Bc	16.6 ± 12.9 ABb	8.3 ± 8.3 Bc
	D4	210.18	63.3 ± 2.7 ABb	78.1 ± 6.61 Aa	37.2 ± 3.9 Bb
	D5	343.85	97.2 ± 2.7 Aa	96.3 ± 2.5 Aa	88.5 ± 11.5 Aa

* D1, natural phenolic dose found in dry plant tissue samples of cover crop plants (except for ferulic acid D1 = 1/2 of concentration found in dry plant tissue samples of cover crop plants) D2 = 2 × D1. D3 = 4 × D1. D4 = 8 × D1. D5 = 16 × D1. ** Ferulic acid was not applied at the highest concentration because it could not be dissolved in distilled water. Values with different lowercase letters (a–c) for the same phenolic acid or with different uppercase letters (A–F) between different phenolic acids differ significantly based on Tukey’s least significant difference test (*p* < 0.05).

**Table 2:** Analysis of variance of variables measured in bioassay.

Source of Variability	N-1	Inhibition Rate (%)		
		Shoot	Radicle	Biomass
Phenolic acids	9	<0.001	<0.001	<0.001
Dose level (Phenolic acids)	40	<0.001	<0.001	<0.001
Residual	100	114.32	181.85	112.57

## Data Availability

All generated data are included in this communication.
